# IMPACT OF PROVIDING IPADS TO OLDER ADULTS: LESSONS CALIFORNIA FUNDED PROGRAM

**DOI:** 10.1093/geroni/igae098.3856

**Published:** 2024-12-31

**Authors:** Sheila Salinas Navarro, Mengzhao Yan, Sindy Lomeli, Carly Roman, Kathleen Wilber

**Affiliations:** University of Southern California, Los Angeles, California, United States; University of Southern California, Los Angeles, California, United States; University of Southern California, Los Angeles, California, United States; Archstone Foundation, Long Beach, California, United States; University of Southern California, Los Angeles, California, United States

## Abstract

The California Department of Aging innovative Connections, Health, Aging, & Technology (CHAT) program has bridged the digital divide by providing free iPads with internet access to older adults aged 60 and over. This initiative sought to enhance mental and physical well-being through increased community engagement, information, and access to services. Our research employed a mixed-method approach to understand participants’ usage and motivations. We used Stata 18 to analyze data from 980 participants who completed a pre and post-test. Most used their iPads more than once a day (n=454; 46%) or once a day (n=211; 22%); 62 participants (6%) did not use the iPad. In a qualitative analysis conducted with Dedoose using grounded theory, we identified key themes for using the iPad, including the role of pre-test expectations and motivators identified in the post-test. Initially, participants anticipated using their iPads for communication, learning, well-being, technology, and simplifying life. However, post-test results revealed a fascinating shift in motivations, now centered on technology, education, entertainment, communication, and health. This shift underscores the profound impact of the CHAT program on the participants’ attitudes and behaviors. Understanding what motivates users is crucial for tailoring programs that support older adults in mastering advanced features and enhancing their connection to loved ones, information, the community, and healthcare providers. Our findings suggest that older adults are not only interested in but also actively engaged with technology for a variety of activities.

